# Nonepisodic Angioedema with Eosinophilia

**DOI:** 10.31662/jmaj.2021-0096

**Published:** 2021-09-13

**Authors:** Thatchai Kampitak

**Affiliations:** 1 Allergy Unit, Department of Medicine, Samitivej Sukhumvit Hospital, Bangkok, Thailand

**Keywords:** angioedema, eosinophilia, nonepisodic

A previously healthy 46-year-old Japanese man presented with a few-day history of bilateral symmetrical swelling of the lower extremities following mild influenza-like symptoms 1 week prior (Figure 1A). Only markedly high eosinophilia count (4,760/mm^3^) was found on laboratory tests. He was diagnosed with nonepisodic angioedema with eosinophilia (NEAE), which spontaneously resolved after 2 months (Figure 1B).

**Figure 1.**
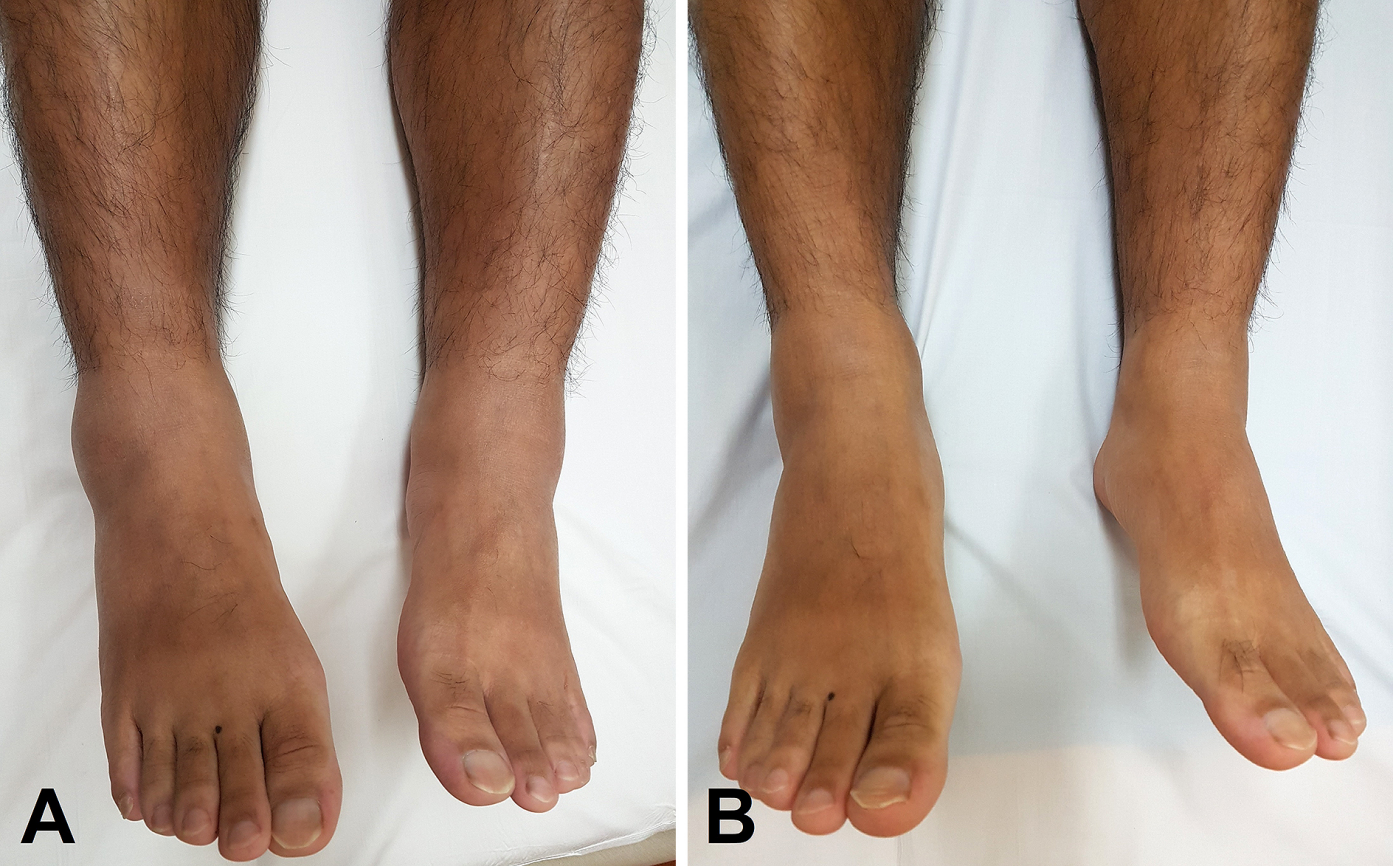
Bilateral symmetrical swelling of the lower extremities in a 46-year-old Japanese man due to nonepisodic angioedema with eosinophila; at presentation (A) and at resolution (B).

Nonepisodic angioedema with eosinophilia is characterized by nonrecurrent peripheral angioedema with eosinophilia and normal IgM levels along with lack of fever, weight gain, and internal organ involvement. It mainly affects young women from Japan, Korea and Thailand ^(1), (2), (3), (4)^. NEAE in men or involving body parts other than the lower extremities is unusual ^(5)^. Although its exact pathophysiology remains unknown, the female predominance and occurrence of this disease following infection or drug exposure in some patients are suggestive of NEAE being a consequence of an aberrant immune response to various exogenous stimuli in genetic susceptible individuals under the influence of sex hormones ^(4)^. Corticosteroid therapy is generally reserved for patients with severe symptoms or marked eosinophilia. However, regardless of treatment, even the most affected patients completely recover within few months after presentation.

## Article Information

### Conflicts of Interest

None

### Author Contributions

Thatchai Kampitak contributed to manuscript preparation, patient care, and discussion

### Approval by Institutional Review Board (IRB)

IRB approval was not required for this study

### Informed Consent

Informed consent has been obtained from the patient
